# Editorial: The biological mechanism and health effect of co-infection with multiple pathogens

**DOI:** 10.3389/fcimb.2024.1370067

**Published:** 2024-01-31

**Authors:** Li Dong, Li Xing

**Affiliations:** ^1^ Institutes of Biomedical Sciences, Shanxi University, Taiyuan, Shanxi, China; ^2^ Shanxi Provincial Key Laboratory of Medical Molecular Cell Biology, Shanxi University, Taiyuan, China; ^3^ Shanxi Provincial Key Laboratory for Prevention and Treatment of Major Infectious Diseases, Taiyuan, China; ^4^ The Key Laboratory of Chemical Biology and Molecular Engineering of Ministry of Education, Shanxi University, Taiyuan, China

**Keywords:** co-infection, HIV, Moraxella catarrhalis, Klebsiella pneumoniae, *Mycobacterium tuberculosis*

Co-infection by multiple pathogen species (*i.e.* viruses, bacteria, protozoa, fungal parasites, helminths et al.) is a ubiquitous phenomenon for infectious diseases, but the clinical outcomes and underlying mechanisms are complicated. HIV infection, tuberculosis, malaria (Boraschi et al., 2008), hepatitis (Bosh et al., 2018), leishmaniasis (Graepp-Fontoura et al., 2023) and respiratory infections (Liu et al., 2021) are always involved in co-infection globally. Co-infecting pathogens affect each other in multiple ways, such as competing for essential resources for living (Wale et al., 2017), interfering with replication, or indirectly interacting against the host immune system (Ezenwa et al., 2010), which can subsequently alter the pathogens transmission mode, disease aggressiveness and clinical symptoms and outcome. The status of co-infected patients can be complexed by overlapping drug toxicities and interactions, which challenges the optimal therapeutic regime. Co-infection at the population level can change the epidemic dynamics and determine the severity (Susi et al., 2015). For example, secondary bacterial co-infection following infection of influenza viruses (Shrestha et al., 2013), respiratory syncytial virus (RSV) (Lin et al., 2022), or severe acute respiratory syndrome coronavirus 2 (SARS-CoV-2) (Antuori et al., 2023) contribute significantly to the loss of lives. So far, both experimental and mathematical models were applied to explore the mechanisms underlying the diverse clinical outcomes of co-infections. A number of factors including orders of pathogen arrival (Clay et al., 2020), timings, and pairing may cooperatively or synergistically determine disease severity (Pinky et al., 2023). The clinical needs and complexity of interactions between co-infecting pathogens necessitate further recording and analysis of co-infection under different circumstances. Herein, we still call the basic and clinical research papers focusing on the mechanism and consequences of virus or other pathogens co-infection, providing insightful information to facilitate the development of infectious disease treatment.

The current edition of Frontiers in cellular and infection microbiology features five articles highlighting the Rhinovirus (RV), HIV, Klebsiella pneumoniae, Pseudomonas aeruginosa, and Acinetobacter baumanii-involved co-infections, respectively. One USA-based study used a co-infection model of human airway epithelium to test the effects of Rhinovirus infection on the existence of Moraxella catarrhalis. Another USA-based article describes the risk factors for co-infection with carbapenem-resistant Klebsiella pneumoniae and carbapenem-resistant Pseudomonas aeruginosa or Acinetobacter baumanii. Three articles address HIV-involved co-infections, where one cohort study was done by cooperation of Brazil and South Africa scientists to present the correlation of anemia and mycobacterial dissemination with the mortality of TB-associated HIV; one Chinese study explored the clearance of hepatitis B surface antigen during combined antiretroviral therapy of HIV/HBV (hepatitis B virus) co-infection; and one article reported a clinical case co-infected with HIV, HBV, HCV, and Vibrio vulnificus. Moraxella catarrhalis (M. catarrhalis) is causally associated with otitis media, sinusitis, and other chronic obstructive pulmonary disease. The abundance of Moraxella catarrhalis was observed previously to be increased during RV infection, but the mechanisms are still not clear. Dissanayake et al. established an *in vitro* model of differentiated bronchial epithelial cells and determined the effects of RV co-infection on M. catarrhalis cell association, abundance, and cellular responses. They inoculated RV-A16 to the apical surface of differentiated bronchial epithelium, and 2 hours later a clinical isolate of M. catarrhalis (strain MC14) was added. They found that RV-A16 infection caused significant cytotoxicity as indicated by LDH release and significantly increased cell-associated M. catarrhalis, but M. catarrhalis did not influence RV replication. These observed effects were specifically associated with the virulent RV strains (e.g. RV-A and RV-C types) (Lee et al., 2012), and appeared due to the RV-A16 infection-induced increase of expression level of carcinoembryonic antigen-related cell adhesion molecule (CEACAM), a key cellular receptor for outer membrane proteins UspA1 and UspA2 of M. catarrhalis. The results added new evidence and shed light to the previous notion that viral infection made the population susceptible to the secondary bacterial burden (Shrestha et al., 2013; Lin et al., 2022).

By comparatively analyzing 86 patients with carbapenem-resistant Klebsiella pneumoniae (CRKP) mono-infection and 60 patients with co-infections of additional carbapenem-resistant Pseudomonas aeruginosa (CRPA) or Acinetobacter baumannii (CRAB), Sophonsri et al. reports that respiratory tract instead of urinary tract was the predominant infection site for co-infected patients. Compared with CRKP mono-infection, co-infected patients usually have prior carbapenem exposure and history of pneumonia in the past year. Moreover, coinfected patients often required direct intensive care unit (ICU) admission with a prolonged length of stay (median 15 vs 10 days). These results are informative for physician to formulate the checking and therapeutic treatment strategy.

The remaining three articles described the HIV-related co-infections and are especially meaningful for clinical practice. Araújo-Pereira et al. studied 496 hospitalized adult people living with HIV (PLHIV) with CD4 count <350 cells/μL and high clinical suspicion of new tuberculosis (TB) infection enrolled in Cape Town (South Africa) between 2014-2016 and found that patients with severe anemia exhibited greater systemic inflammation, high levels of IL-8, IL-1RA and IL-6, which were associated with a higher *Mycobacterium tuberculosis* (*Mtb*) dissemination score and a higher risk of death. Li et al. performed a multivariate logistic analysis of 51 patients with HIV/HBV co-infections after initiating combined antiretroviral therapy (cART) and revealed that lower baseline CD4+ T cell levels (OR=6.633, P=0.012) and soluble programmed death-1 (sPD-1) level (OR=5.389, P=0.038) were independently associated with earlier rapid clearance of hepatitis B surface antigen (HBsAg) after cART initiation, which was also associated with the higher alanine aminotransferase abnormality rate and higher level of immune activation marker HLA-DR. Zeng et al. described diagnostic characters and successful treatment of a 48-year-old man co-infected with Vibrio vulnificus and multiple viruses including HIV, Hepatitis A and hepatitis B from Guangdong’s coastal region in October, 2022. Instead of performing an amputation, the Vibrio vulnificus-infected limb was recovered by regular dressing changes, thorough debridement, wound closure, ongoing vacuum-sealing drainage (VSD), and local antibiotic irrigation. Taken together, these three articles provide novel perspectives on HIV-related co-infections and contribute to a better precisive diagnosis and treatment to improve clinical care of co-infected patients.

## Author contributions

LD: Writing – original draft, Writing – review & editing. LX: Conceptualization, Writing – original draft, Writing – review & editing.
